# Equal Efficacy and Safety Profile in Elderly Patients with Hepatocellular Carcinoma Receiving Palliative Treatment

**DOI:** 10.3390/cancers14030768

**Published:** 2022-02-01

**Authors:** Thorben W. Fründt, Christian Casar, Johann von Felden, Ulrike Schöler, Maximilian Priebe, Jenny Kraczyk, Hannes Ahrend, Johannes Salamon, Gerhard Adam, Samuel Huber, Ansgar W. Lohse, Henning Wege, Kornelius Schulze

**Affiliations:** 1 I. Department of Medicine, Gastroenterology and Hepatology, University Medical Center Hamburg-Eppendorf, 20246 Hamburg, Germany; j.von-felden@uke.de (J.v.F.); sch-ulrike@web.de (U.S.); maximilianpriebe@t-online.de (M.P.); s.huber@uke.de (S.H.); a.lohse@uke.de (A.W.L.); h.wege@klinikum-esslingen.de (H.W.); k.schulze@uke.de (K.S.); 2 Bioinformatics Facility, University Medical Center Hamburg-Eppendorf, 20246 Hamburg, Germany; c.casar@uke.de (C.C.); jenny.kraczyk@uke.de (J.K.); 3 Department of Internal Medicine, Israelitic Hospital, 22297 Hamburg, Germany; h.arhrend@ik.de; 4 Department of Diagnostic and Interventional Radiology and Nuclear Medicine, University Medical Center Hamburg-Eppendorf, 20246 Hamburg, Germany; j.salamon@uke.de (J.S.); g.adam@uke.de (G.A.); 5 Cancer Center Esslingen, Klinikum Esslingen, 73730 Esslingen am Neckar, Germany

**Keywords:** hepatocellular carcinoma, elderly patients, sorafenib, TACE, palliative treatment

## Abstract

**Simple Summary:**

Hepatocellular carcinoma (HCC) is the most common primary liver tumor and a leading cause of cancer-related death worldwide with an increasing incidence, especially in elderly people. However, as elderly patients are often characterized by other comorbidities and frailty, palliative treatment in this subgroup of patients remains challenging for clinicians. In this retrospective study, we found a good tolerability and safety of elderly patients receiving palliative treatment for metastatic HCC, primarily transarterial chemoembolization and systemic treatment. Furthermore, we found no significant difference in terms of overall survival between younger and older patients in this cohort.

**Abstract:**

Palliative treatment of elderly patients with hepatocellular carcinoma (HCC) is often challenging due to comorbidities or frailty, and data about the outcome and overall survival (OS) in these patients are limited. This was a retrospective single centre study. Patients were grouped according to their age as young (<60 years; YP), intermediate (60–70 years; IP) or elderly (>70 years; EP). Administration of chemotherapy or transarterial chemoembolization (TACE) was defined as palliative treatment. Therapy-related adverse events (AE) were assessed via CTCAE 5.0. Out of 656 patients analyzed, *n* = 359 received palliative treatment: YP: *n* = 90; IP: *n* = 127 and EP: *n* = 142. The median OS (months) in patients receiving TACE (*n* = 254) was 17 vs. 18 vs. 20 months for YP, IP, and EP, respectively (*p* = 0.44) and 15 vs. 16 vs. 17 months (*p* = 0.56), respectively, in patients receiving chemotherapy (*n* = 105). AEs differed non-significantly between the subgroups. Multivariate analysis revealed impaired liver function and advanced tumor stage as significant factors for impaired OS. In this study, the mOS and rate of AEs were equal between elderly and younger HCC patients receiving palliative treatment. Therefore, we propose regular palliative treatment stratification in spite of the high age of patients.

## 1. Introduction

Hepatocellular carcinoma (HCC) is the most common primary liver tumor and the second most common cause of cancer-related death worldwide [1,2]. Liver cirrhosis due to chronic viral infection (e.g., chronic hepatitis B or hepatitis C virus infection) or non-viral liver diseases (non-alcoholic steatohepatitis (NASH) or alcoholic liver disease) is the main risk factor for tumor development. Despite recent achievements in the treatment of chronic hepatitis C (HCV) infection, the incidence of HCC is constantly increasing, mainly due to an increase in NASH-related liver disease, obesity and type two diabetes in the western population [3]. For example, the incidence rate of liver cancer tripled between 1975 to 2011 in the United States, leading to an age-adjusted incidence of 8.6 per 100,000 people [4]. Except for hepatitis B virus (HBV)-related liver cirrhosis, where infection mainly occurs through vertical transmission, liver cirrhosis due to HCV or non-viral liver diseases primarily develops in adults. Because the risk for cancer development increases over time, HCC in these patients is often diagnosed in middle or advanced age [5]. In fact, a recent study by El-Sarag reported an age-specific HCC increase in patients older than 75 years [6]. However, as elderly patients are often at an increased risk for other comorbidities such as diabetes and cardiovascular or pulmonary disease, it is challenging for clinicians to define the optimal treatment strategy in this cohort [7,8,9,10]. This challenging constellation is further complicated by the fact that the diagnosis of HCC is often made at an intermediate or advanced tumor stage, and the recommended treatment according to recent guidelines consisted of transarterial chemoembolization (TACE) or palliative systemic therapy, both associated with relevant side effects [11,12,13]. Despite a rapid change in the landscape of HCC treatment regimens during the last few years with the introduction of new agents to the market such as the tyrosine kinase inhibitors (TKI) lenvatinib, the VEGF 2-receptor antibody ramucirumab and, finally, anti PD-1 antibodies, resulting in improved treatment options in HCC patients, the implication of this achievement in older patients still is unclear, as predominantly younger patients in a good clinical status have been included in the relevant phase III trials [14,15,16,17]. Although older age is a risk factor for cancer itself, the prognostic impact of age in patients with solid tumor is controversial: while a poor prognosis for older patients has been reported for prostate and thyroid cancer, in patients with colon or breast cancer the outcome was beneficial in comparison to younger patients [18,19,20,21]. However, Guo et al. recently demonstrated that HCC patients at the age of 65 years or older were less often effectively treated with surgery and loco-regional treatment compared to younger patients [22]. Because little is known about the clinical course of elderly HCC patients receiving a non-curative treatment, we conducted a retrospective single centre study at a university medical centre to determine the age-specific clinical characteristics, safety and tolerability of palliative treatment and outcome in HCC patients.

## 2. Materials and Methods

### 2.1. Study Population

A total of 987 patients with confirmed diagnosis of HCC who were treated at the University Medical Centre Hamburg-Eppendorf between 2008 and 2017 were included in this study. Electronic patient files were reviewed to assess tumor stage at diagnosis, baseline demographic characteristics and laboratory values, treatment modalities and clinical course. The follow-up period was determined based on the date when diagnosis of the tumor was made until patient’s death or last documented patient contact with the outpatient clinic. The World Health Organization (WHO) definition of elderly is a person 65 years or older, but this implies a rather heterogeneous group of patients. Apart from the WHO definition, there is no consensus definition of older age in the literature and different thresholds for elderly patients have been used in previous studies. Therefore, to provide a more differentiated and precise classification, we defined patients younger than 60 years as young patients (YP), patients at the age of 60 to 70 years as intermediate age patients (IP) and patients older than 70 years as elderly patients (EP).

**Figure 1:**
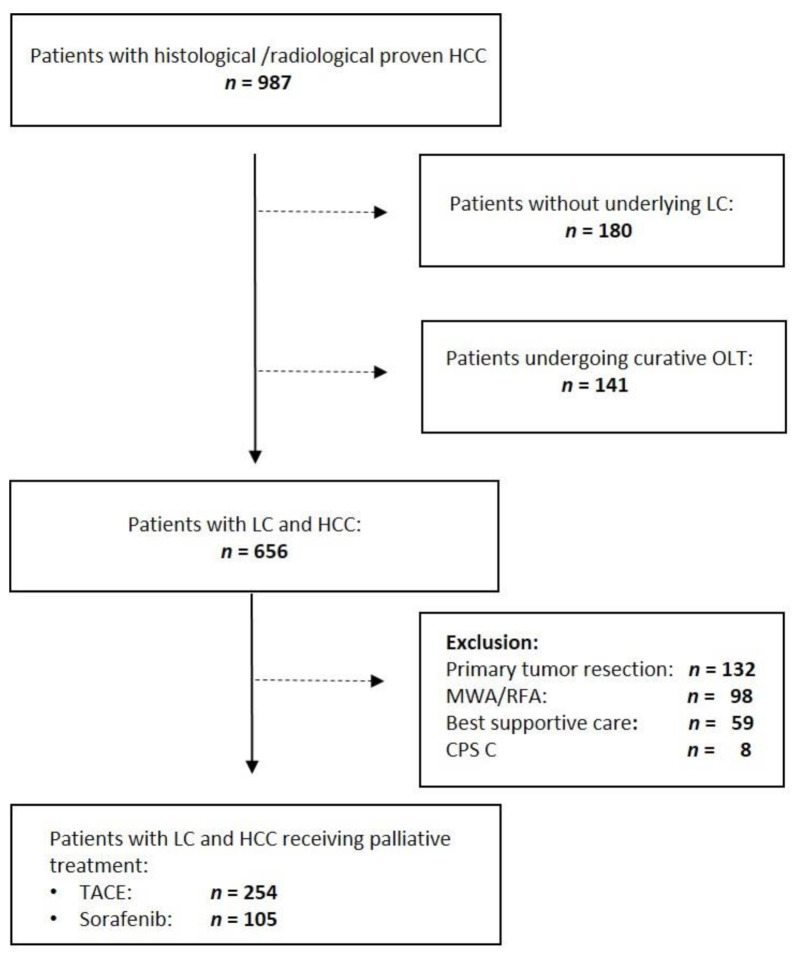
Flow chart of the study. Out of 987 patients analyzed, *n* = 321 were excluded because they had no underlying liver cirrhosis or had undergone orthotopic liver transplantation (OLT; *n* = 141). Out of the remaining 656 patients, *n* = 359 were included in the final analysis. CPS, Child–Pugh score; LC, liver cirrhosis; MWA, microwave ablation; RFA, radiofrequency ablation.

In order to ensure comparability of data, only patients with underlying liver cirrhosis were included in this study. Diagnosis of HCC was confirmed either histologically or radiologically by imaging criteria (e.g., by computed tomography (CT) scan or magnetic resonance imaging (MRI)) according to recent guidelines. Tumor extent was assessed according to the Barcelona Clinic Liver Cancer (BCLC) classification and patients liver function was rated using the Child–Pugh Score (CPS). Eastern Cooperative Oncology Group (ECOG) performance score was used to assess patient’s function status. All patients were discussed at a multidisciplinary conference to determine treatment modalities: treatment options included surgical resection, microwave (MWA) or radiofrequency ablation (RFA) and orthotopic liver transplantation for patients with BCLC stage 0 or A. All eligible patients, who underwent OLT (*n* = 141), MWA/RFA (*n* = 98) or tumor resection (*n* = 132) were excluded from further analysis, as parameters influencing overall survival (OS; e.g., liver function of underlying liver cirrhosis, susceptibility) changed gradually at the time of transplantation or curative intended treatment. Additionally, cirrhotic patients with CPS C, who had exceptionally received palliative treatment (*n* = 8) in a compassionate use, were excluded from final analysis because treatment of this subgroup of patients with deteriorate liver function is not recommended in recent guidelines. In total, *n* = 367 patients were included in the final analysis (Figure 1).

Treatment modalities for patients in a palliative stage included transarterial chemoembolization (TACE) and, for patients with distant metastases or macro vascular invasion of the tumor, palliative systemic therapy. Treatment-related adverse events (AE) were rated according the Common Terminology Criteria for Adverse Events (CTCAE) v5.0 [23].

### 2.2. Transarterial Chemoembolization

TACE was carried out in the Department of Diagnostic and Interventional Radiology, University Medical Center Hamburg-Eppendorf, Germany, by experienced interventional radiologists. Following the puncture of the femoral arteria, a sidewinder catheter was inserted and placed in the hepatic artery, and an angiography was performed to evaluate the vascular structure and to precisely localize the tumor. Following the angiography, a mixture of doxorubicine and lipidol or drug-eluting beads (DEB) was injected into the tumor-feeding artery until enrichment of the embolization mixture in the tumor lesion was visible. Response to TACE was evaluated via contrast-enhanced CT scan six to eight weeks after the procedure.

### 2.3. Systemic Therapy

For systemic palliative therapy, mainly the TKI sorafenib at a dose of 200 mg per tablet had been used. Sorafenib was orally administered with a maximum dose of 800 mg per day, divided in two doses per day (b.i.d). Furthermore, but only in a very small number of patients, capecitabine (orally administered at a dose of 1000 mg per m^2^ of body surface b.i.d. for 14 days of 21 day circle), everolimus (orally administered 7.5 mg per day), tivantinib (120 mg or 240 mg b.i.d) and doxorubicine (administered intravenous at a dosage of 30 mg or 20 mg per m^2^ body surface on day 1, treatment was repeated every four weeks) had been used for systemic treatment, primarily in patients with contraindications to sorafenib or in a compassionate use.

### 2.4. Statistical Analysis

Categorical variables were described in terms of percentages and frequencies; continuous variables were described in terms of median with min/max range. Media OS was compared using Kaplan–Meier curves with the log-rank test. Fisher’s exact test was used to compare distribution of count data between groups. Kruskal–Wallis rank sum test was used to compare continuous variable. Chi-square test was used to compare categorical variables. Cox regression analysis was used for multivariable testing. For all outcomes received, a *p*-value < 0.05 indicated statistical significance. All statistical analyses were conducted using R, version 4.1.

### 2.5. Ethical Approval

Data were anonymized and analysis could thus be conducted in accordance with local government law (Hamburgisches Krankenhausgesetz §12, accessible: https://www.landesrecht-hamburg.de/bsha/document/jlr-KHGHAV7P12, accessed on 12 November 2021).

## 3. Results

Out of the 656 patients analyzed, *n* = 194 patients were classified as young (YP, age between 18 and 60 years), *n* = 241 as intermediate age (IP, 60–70 years) and *n* = 221 as elderly patients (EP, ≥70 years). The main etiology of LC was alcoholic liver disease (37.5%), followed by chronic HCV and HBV infection (21% and 13%, respectively) in the entire cohort. The distribution of LC etiology differed significantly between YP, IP and EP with a larger proportion of HCV- (32.2% vs. 18.5% vs. 15.9%, respectively) and HBV-related LC (21.2% vs. 11.6% vs. 6.7%, respectively; *p* = 0.005) in the YP subgroup. In this subgroup, patients had significantly more often an impaired liver function with a higher CPS, while in the EP group, the rate of patients with deteriorated liver function was the lowest (CPS A, B, C: 55.3%, 28.5%, 28.2% vs. 61%, 26.5%, 12.5% vs. 66%, 28%, 6%, respectively; *p* = 0.01). The number of intrahepatic tumor lesions and median level of alpha-fetoprotein (AFP) level at diagnosis were comparable between the groups. The baseline characteristics are depicted in Table 1.

Out of the 656 patients included in this study, palliative treatment was administered in *n* = 381 patients: a total of *n* = 254 patients underwent TACE, while *n* = 119 patients received systemic therapy (sorafenib: *n* = 105; everolimus: *n* = 8, doxorubicin: *n* = 4, tivantinib: *n* = 1, capecitabin: *n* = 1).

**Figure 2:**
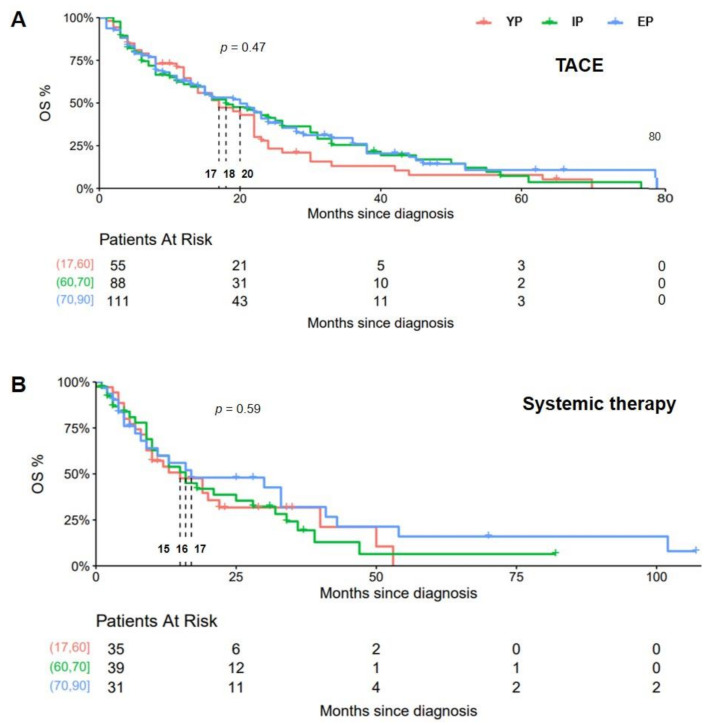
Kaplan–Meier analysis patients undergoing palliative therapy grouped according to their age and type of therapy. Out of *n* = 656 patients analyzed, *n* = 254 underwent TACE and *n* = 105 received sorafenib. In the TACE cohort (**A**) and in the sorafenib subgroup (**B**), mOS differed non-significantly between the age-defined subgroups.

The median overall survival (mOS) differed non-significantly in the cohort of patients receiving TACE (17 vs. 18 vs. 20 months, respectively; *p* = 0.47) and in patients undergoing systemic therapy (15 vs. 16 vs. 17 months, respectively; *p* = 0.59). Kaplan–Meier analysis is depicted in Figure 2. Regarding tumor stage, the mOS was without significant difference between YP, IP and EP in patients with an intermediate tumor stage (BCLC B; 15 vs. 16 vs. 18 months, respectively; *p* = *0*.44), but a trend toward prolonged mOS for IP in the cohort of patients with progressed disease was found (BCLC C; 5 vs. 10 vs. 8 months, respectively; *p* = 0.054).

**Figure 3:**
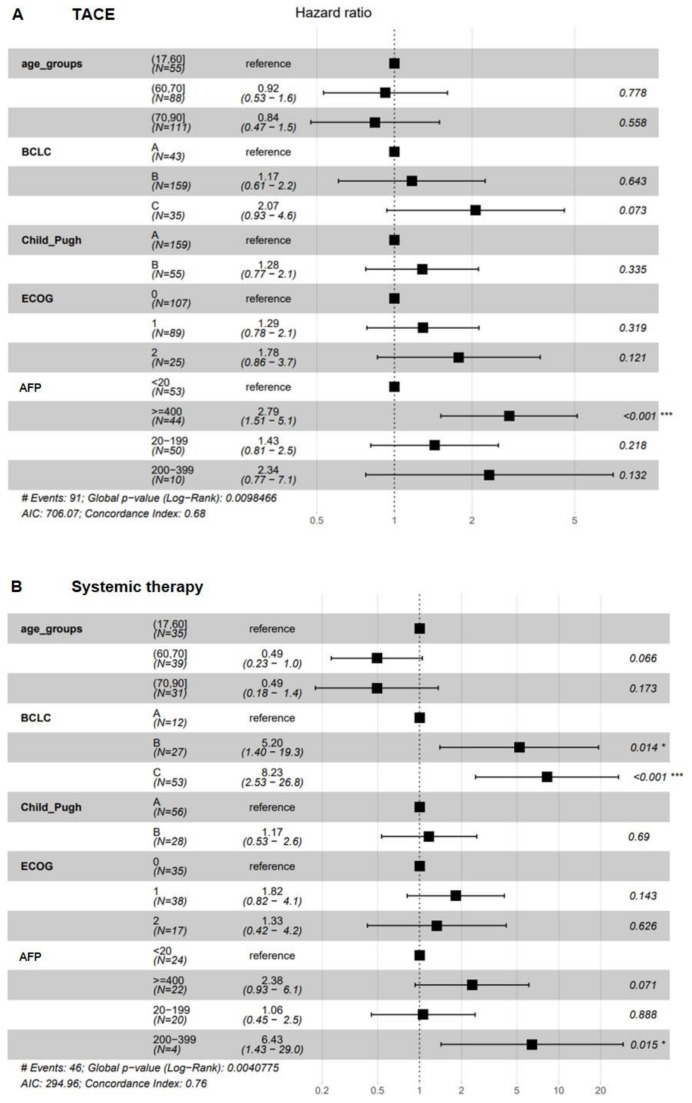
COX regression analysis. Elevated AFP was significantly associated with impaired overall survival in patients undergoing TACE (**A**). Among the patients who had received systemic therapy, multivariate testing identified advanced tumor stages (BCLC B and C) and elevated AFP as significant risk factors for impaired OS (**B**). Higher age was not associated with reduced survival, neither in TACE nor in the sorafenib cohort. Significance level: * *p* < 0.05; *** *p* < 0.001.

In a Cox regression analysis, an AFP level > 400 ng/mL (HR: 2.79; 95% CI: 1.5–5.1; *p* < 0.001) were associated with significantly reduced OS in patients undergoing TACE (Figure 3A). For patients receiving systemic treatment, multivariate analysis identified the tumor stage (BCLC B: HR: 5.2, 95% CI: 1.40–19.3; *p* = 0.014; BCLC C; HR: 8.23; 95% CI: 2.53–26.8; *p* = < 0.001) and an AFP level between 200–399 ng/mL (HR: 6.43, 95% CI: 1.43–29.0; *p* = 0.015) as risk factors for impaired survival (Figure 3B).

**Figure 4:**
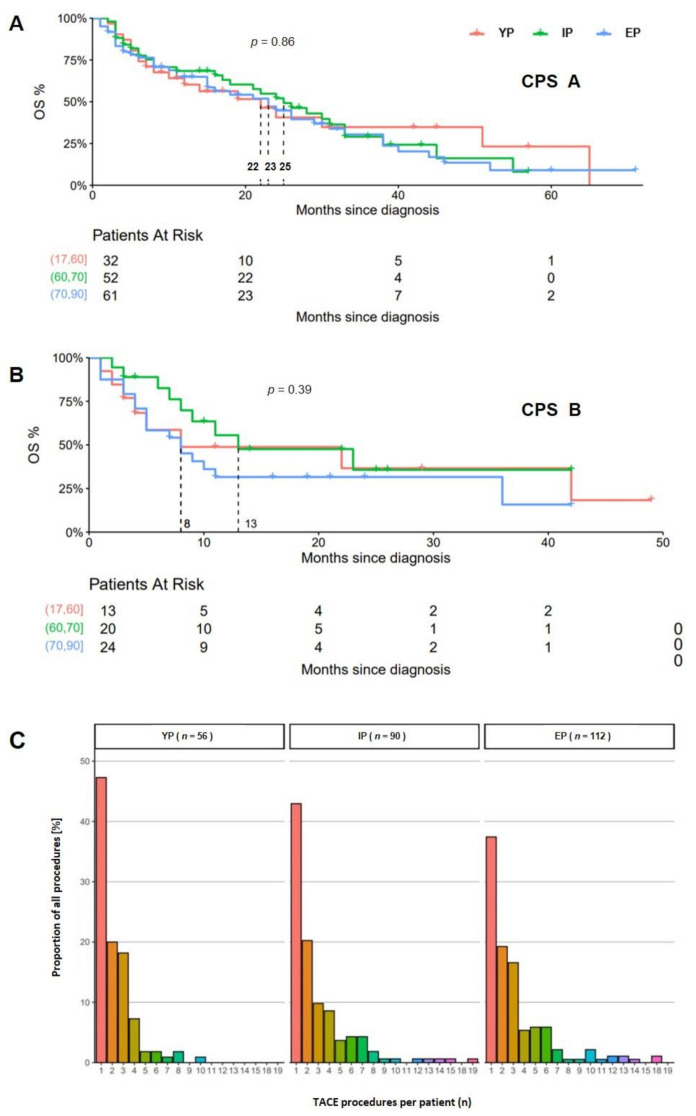
Kaplan–Meier analysis of patients undergoing TACE. In patients with preserved liver function CPS A (**A**), mOS ranged between 22 to 25 month. In the subgroup of patients with liver cirrhosis CPS B, mOS decreased to 8 months in the YP and EP cohort (**B**). In all 258 patients, TACE was carried out at a median of *n* = 2 in YP, *n*= 3 in IP and *n* = 4 in EP (**C**).

In a subgroup analysis regarding liver function, the mOS in the TACE cohort was (YP, IP, EP) 22 vs. 23 vs. 25 months, respectively, (*p* = *0*.86) in patients with liver cirrhosis CPS A and 8 vs. 13 vs. 8 months, respectively, (*p* = *0*.39) in CPS B patients (Figure 4).

**Figure 5:**
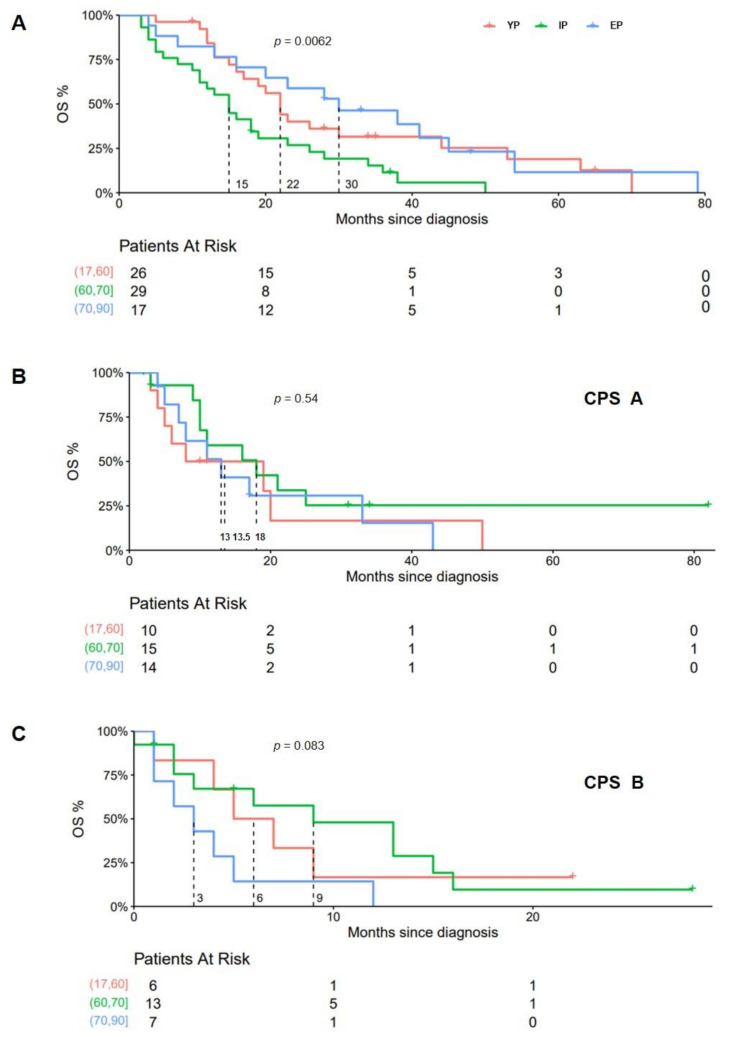
Kaplan–Meier analysis of patients receiving palliative systemic therapy. In patients who had initially undergone TACE and were then switched to systemic therapy due to tumor progression, median OS was significantly longer in EP than YP and IP (**A**). For patients who had been started on sorafenib at first diagnosis, median OS differed non-significantly in the subgroup of CPS A (**B**) and CPS B (**C**) but was found markedly decreased in patients with impaired liver function.

In patients who had received systemic therapy, mOS in CPS A patients was (YP, IP, EP) 13 vs. 18 vs. 13.5 months (*p* = 0.54) and 6 vs. 9 vs. 3 months, respectively, (*p* = 0.083) in CPS B (Figure 5). Seventy-two patients had been treated with sorafenib following tumor progression after TACE (sequential treatment). In this cohort, mOS was significantly prolonged in EP (22 vs. 15 vs. 30 months, respectively; *p* = 0.0062) (Figure 5A).

**Figure 6:**
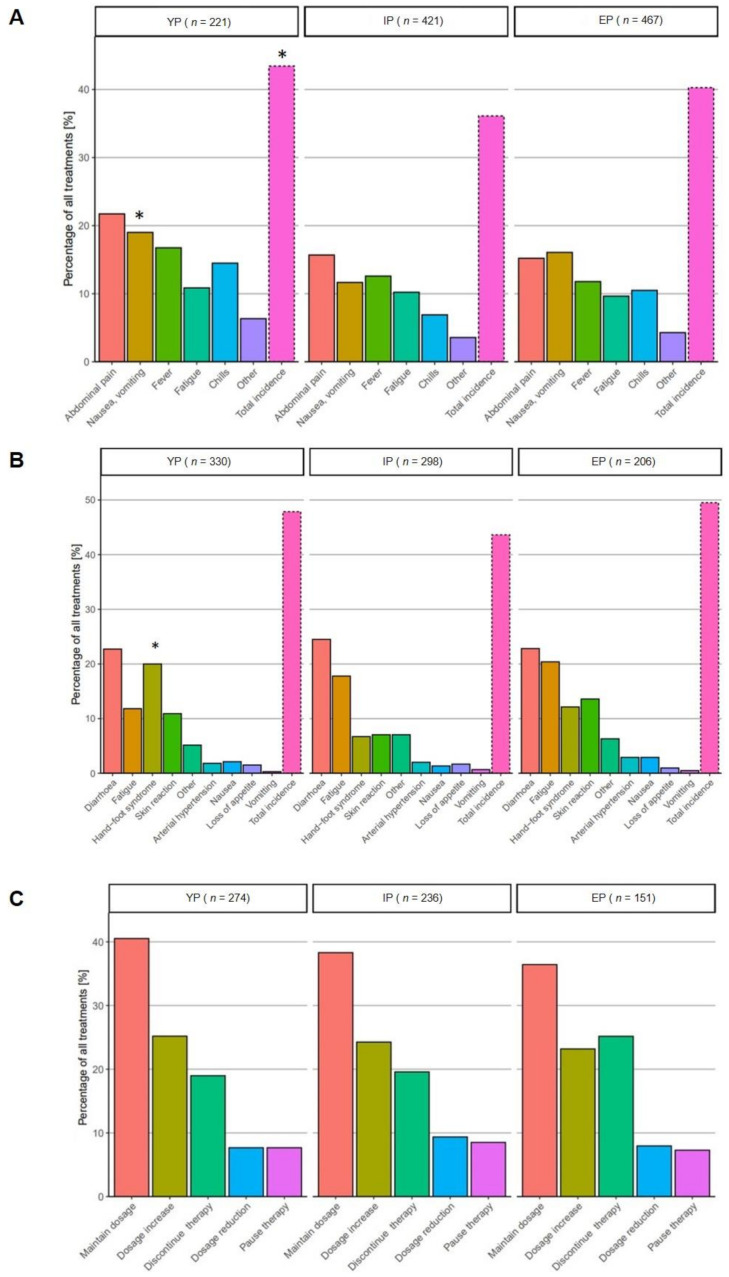
Treatment-related adverse events in patients receiving palliative therapy. In patients who had undergone TACE, a total of *n* = 797 presentations to the outpatient clinic had been analyzed (number of visits (*n*) are depicted at the top of every column): overall incidence of treatment-related AEs was significantly higher in YP (43% vs. 36% vs. 40%, respectively; *p* = 0.021), in particular, nausea, vomiting, chills and abdominal pain developed significantly more often in this subgroup (**A**). While the total incidence of AEs in sorafenib-treated patients differed non-significantly (48% vs. 44% vs. 50%, respectively; *p* = 0.21), hand–foot syndrome developed significantly more often in YP (17% vs. 5% vs. 14%, respectively; *p* < 0.005) (**B**). In the subgroup of patients receiving sorafenib, the rate of dose modification in terms of dose reduction (7.6% vs. 9% vs. 7.9%, respectively; *p* = 0.23), pausing (7.6% vs. 8.5% vs. 7.3%, respectively; *p* = 0.21) or discontinuing treatment (19% vs. 19.6% vs. 25%, respectively; *p* = 0.67) was comparable in all three subgroups, whereas maintenance of dosage was found significantly more often in IP cohort (**C**). Significance level: * *p* < 0.05.

Treatment-related AEs and rate of dose modifications among the subgroups is depicted in Figure 6. In patients treated with TACE, total incidence of AE differed significantly between the three groups (43.4% vs. 36% vs. 40.3%, respectively; *p* = 0.02); nausea and vomiting was found significantly more often in YP (19% vs. 11.6% vs. 16%, respectively; *p* = 0.04), while incidence of fever, chills and fatigue was comparable in each group.

## 4. Discussion

The incidence of HCC is increasing worldwide, and the prevalence of first diagnosis is shifting towards an older age and varies according to different countries: the mean age of initial HCC diagnosis ranges between 63 and 65 years for European and North American patients [24,25]. However, despite this global trend, there are only a few studies addressing the outcome and clinical course of elderly patients with HCC.

In this real-life study, we demonstrated that both survival and treatment-related AEs do not differ between younger and elderly patients. Furthermore, in our analysis, tumor age according to the BCLC system and the tumor marker AFP were relevant risk factors for survival, whereas higher age was not associated with impaired survival, either in the cohort of patients who had undergone TACE or in the subgroup of patients receiving systemic therapy.

In our cohort of HCC patients we found that the etiology of underlying liver cirrhosis differed markedly between YP, IP and EP: while chronic hepatitis infection was found significantly more often in younger patients, EP were more likely to have a liver cirrhosis due to alcoholic liver disease or non-viral etiology. These findings are in line with the study of Oishi et al. who reported a significantly lower rate of chronic viral infections in elderly HCC patients presenting for hepatectomy [26]. Furthermore, elderly HCC patients were found to have more likely chronic HCV than HBV infection because HBV is often transmitted perinatally, leading to the development of liver cirrhosis and, subsequently, HCC at a younger age [27]. In fact, with increasing age, we found a relative decrease in chronic HBV infection compared to chronic HCV infection in our cohort.

Elderly patients primarily had a preserved liver function (CPS A), there was a larger proportion with impaired ECOG status (ECOG 1 and 2) and were more often diagnosed in an intermediate tumor stage (BCLC B), while the rate of patients in an advanced stage (BCLC C) differed only slightly between YP, IP and EP. These findings are consistent with previous studies [28,29]. A possible explanation for the more advanced tumor stage at first diagnosis in EP is that next to the tumor extent, the patient’s performance status is incorporated in the BCLC staging system, thus leading to a higher BCLC class in these patients due to the accompanying frailty [29].

According to recent guidelines, TACE is the recommended treatment for patients with unresectable HCC without extrahepatic spread and compensated liver function [13,30]. TACE has been demonstrated to significantly improve OS in HCC patients with a mean OS of 28.6 months and has also been demonstrated to be effective in patients of age 65 or older [31,32,33]. Furthermore, in a recent studies, TACE has been shown to be safe and effective in both younger and elderly patients, with a comparable outcome regarding long-term OS [28,34]. In our study, mOS differed non-significantly between patients of different ages undergoing TACE, and multivariate testing demonstrated a rather reduced hazard ratio for death in elderly patients. In a subgroup analysis, mOS was >20 month in patients with a compensated liver function regardless of age, but Cox regression revealed that impaired liver function is significantly associated with reduced survival. These findings are consistent with the study of Mirici-Cappa et al. [28]. In patients with CPS B, mOS decreased markedly in all age-defined cohorts with the lowest OS of 8 months in YP und EP, further underlining the impact of liver function in cirrhotic patients. Taken together, our findings support the study of Yau et al. and Takayasu et al., who identified elevated CPS (CPS B/C), but not higher age, as a significant risk factor for impaired survival in young and elderly patients undergoing palliative treatment [34,35]. Additionally, our results underline the fact that caution should be raised when initiating palliative treatment in patients with impaired liver function, regardless of age.

Interestingly, in terms of TACE-related side-effects, YP were found to have AEs significantly more often compared to IP and EP. Among all AEs assessed, chills, abdominal pain, nausea and vomiting occurred significantly more often in YP than in the two other cohorts, while the remaining AEs differed non-significantly between the groups, underlining that TACE is a safe and tolerable procedure in elderly patients.

Since the release of the SHARP study in 2008, sorafenib has been the only systemic treatment option with proven benefits for patients with advanced HCC, until the TKI lenvatinib was found to be non-inferior to sorafenib in terms of OS in 2018 [16,36]. In our study, sorafenib was the treatment of choice for BCLC C patients. While mOS for IP and EP in the BCLC C-cohort was comparable to the mOS presented in the SHARP trial, it was decreased in the YP subgroup to 5 months. A similar median OS of only 6.5 months has been reported in the phase III ASIAN PACIFIC trial, a multicenter study including 226 patients with advanced HCC undergoing sorafenib treatment. In this study, median patient age was 52 years, pointing towards a younger age of included patients [37]. Interestingly, in recent meta-analysis comparing the outcome of sorafenib-treated patients in two consecutive time periods, a significant improvement in mOS has been found in recent years, probably related to a better management of treatment-related side effects, enabling maintenance of sorafenib treatment for a longer period [38]. Our study comprises the same time period as assessed in this meta-analysis, but we did not further subdivide our cohort of sorafenib patients in terms of the date of treatment onset and, therefore, we cannot rule out an improvement in OS over time.

With regard to liver function, we found a decreased survival in patients with liver cirrhosis CPS B. In the subgroup of CPS B patients, mOS was lowest in EP with only three months. These findings are in line with the GIDEON trial, reporting a mOS for CPS B patients of 5 months [39]. The discrepancy between the mOS in our study for CPS B patients and the results reported in the SHARP trial is explained by the fact that the vast majority of patients included in the SHARP study had well-compensated liver cirrhosis (CPS A).

German and international guidelines recommend to initiate systemic treatment when tumor progression following TACE is confirmed [13,30]. In two retrospective studies, a significant improvement in mOS was reported for patients who had been switched to sorafenib following tumor progression after TACE, with an mOS ranging from 24.7 to 25.4 months [40,41]. In our study, mOS differed significantly between the three subgroups and ranged from 15 months in the YP subgroup up to 30 months in the EP cohort. A possible explanation for the decreased mOS in YP is that the proportion of patients with impaired liver function was significantly higher in this subgroup when compared to the IP and EP cohorts. Furthermore, as a worsening of liver function in HCC patients undergoing TACE is reported in approximately 40% of patients, the previous TACE therapy might have also caused a further deterioration in liver function in these patients [42].

In patients treated with sorafenib, treatment-related AEs develop in up to 90%, primarily hand–foot syndrome reaction, anorexia, fatigue and diarrhea [37,43]. In a recent study by Reig et al., all but one out of 147 patients who were prospectively enrolled developed AEs after a median time of 56 days [44]. In our cohort, the cumulative rate of all treatment-related AEs ranged between 48% in the EP cohort to 43% in the IP subgroup. While there was no significant difference between the three subgroups regarding the total incidence of AEs, hand–foot syndrome (HFS) was found significantly more often in the YP cohort. Early onset of HFS or skin reaction in patients treated with sorafenib has been described to be associated with a better outcome in terms of OS in HCC patients [44]. However, due to the non-standardized, retrospective character and relatively small number of patients, we have not addressed this question in our cohort.

Dose modification due to treatment-related AEs is reported in up to 30% of patients receiving sorafenib for metastatic HCC [45]. We found a non-significant trend in our study toward a higher percentage of elderly patients discontinuing therapy. However, apart from this, the rate of patients in whom the dosage had to be reduced or treatment had to be paused was comparable between all three subgroups, demonstrating that sorafenib therapy was tolerated even in patients of higher age.

It is of note that this study has several limitations that need to be addressed: First, this is a single center analysis and due to its retrospective character, patient care had not been standardized a priori. For example, in patients who underwent TACE, agents used within the procedure (e.g., doxorubicine plus lipidol or DEB) differed over time as well as the time point of the post-procedural CT scan for further tumor staging, potentially leading to a delay in either continuing TACE or switching to systemic therapy. Second, in patients treated with sorafenib, treatment adjustments (e.g., dosage de- or increase or withholding of treatment) were solely based on the decision of the responsible clinician and not on predefined criteria. On the other hand, it has to be mentioned that the decision to change treatment modalities, to continue TACE or to initiate systemic treatment was made based on discussion in an interdisciplinary tumor board, including experts in the fields of oncology, radiology and surgery, which further objectifies treatment decisions. Furthermore, the size of the different patients’ subgroups in some cases is small, particularly in the Sorafenib-treated cohort, thus limiting the power of the statistical analysis.

The treatment of elderly patients with solid organ tumors is a remarkable challenge for clinicians: on the one hand, older age is often accompanied by frailty and organ dysfunction, hampering the application of chemotherapy. For example, in patients with metastatic colorectal cancer (mCRC), Aparicio et al. reported a rate of 58% of grade 3 and 4 adverse events in elderly patients undergoing palliative chemotherapy, and in mCRC patients receiving Trifluridine/Tipiracil (TAS), hematologic toxicities were found more often in elderly patients [46,47]. On the other hand, elderly patients are often excluded from clinical trials and, therefore, treatment recommendations are lacking in a recent trial investigating the safety and efficacy of a neoadjuvant chemotherapy in patients with pancreatic cancer, only patients between the ages of 18 and 75 were included [48]. In contrast, a meta-analysis by Nieß et al. supports a radical surgical treatment for patients with pancreatic cancer even beyond the age of 75 years, underlying the difficulty to guide treatment decision in this subset of patients [49,50].

Despite the limitations of this study, our data advocate palliative therapy in elderly patients with HCC as we found no increase in toxicity in elderly patients in either the subgroup of TACE patients or the cohort of patients receiving systemic treatment.

## 5. Conclusions

Taken together, in this study, we demonstrated that the mOS in elderly patients undergoing palliative treatment is comparable to those of a younger age and, therefore, neither TACE nor systemic treatment should be withheld in patients of advanced age. Regardless of age, we found that mOS is markedly decreased in patients with impaired liver function, and we identified impaired liver function and an elevated AFP level as relevant risk factors for patients undergoing TACE, underlining the need for a careful assessment of the liver status prior to the initiation of treatment and even during therapy. Especially in elderly patients with deteriorated liver function, the potential benefits and risks of a systemic therapy should be carefully weighed. Interestingly, we found that the sequential treatment of TACE followed by sorafenib after tumor progression significantly improves the OS in elderly patients, indicating that palliative treatment should be continued in elderly patients.

## Figures and Tables

**Table 1:** Demographic and clinical characteristics of cirrhotic patients with HCC.

Characteristics	YP	IP	EP	*p*
*n*	194	241	221	
Age (median; min/max)	56 (23–60)	66 (61–70)	75 (71–87)	
Male (*n*; %)	164 (84.5%)	198 (82.2%)	180 (81%)	0.61
ECOG (*n*; %)				
0	69 (40.83)	76 (36.54)	61 (33.52)	0.85
1	63 (37.28)	85 (40.87)	75 (41.21)	
2	28 (16.57)	29 (13.94)	32 (17.58)	
3	7 (4.14)	14 (6.73)	11 (6.04)	
BCLC (*n*; %)				
A	55 (31.07)	44 (20.18)	38 (19.69)	0.0054
B	54 (30.51)	100 (45.87)	98 (50.78)	
C	46 (25.99)	52 (23.85)	42 (21.76)	
D	22 (12.43)	22 (10.09)	15 (7.77)	
CPS (*n*; %)				
A	88 (53.33)	122 (61)	111 (66.07)	0.01
B	47 (28.48)	53 (26.5)	47 (27.98)	
C	30 (18.18)	25 (12.5)	10 (5.95)	
Etiology of LC (*n*; %)				
ALD	95 (38.78)	136 (49.45)	105 (41.67)	0.0005
HBV	52 (21.22)	32 (11.64)	17 (6.75)	
HCV	79 (32.24)	51 (18.55)	40 (15.87)	
NAFLD/NASH	6 (2.45)	17 (6.18)	24 (9.52)	
Other	13 (5.31)	39 (14.18)	66 (26.19)	
No. of intrahepatic tumor				
lesions (*n*; %)				
1	65 (43.33)	80 (42.33)	77 (44.25)	0.54
2	35 (23.33)	42 (22.22)	33 (18.97)	
3	13 (8.67)	23 (12.17)	28 (16.09)	
>4	37 (24.67)	44 (23.28)	36 (20.69)	
AFP (ng/mL)				
rang	1.21–2,018,171.1	0.968–457,231.896	1.331–249,118.43	0.71
median	106.2	49.4	57.5	

Abbreviations: AFP, alpha-fetoprotein; ALD, alcoholic liver disease; BCLC, Barcelona Clinic Liver Cancer, CPS, Child–Pugh score; ECOG, Eastern Cooperative Oncology Group; HBV, hepatitis B virus; HCV, hepatitis C virus; NAFLD, non-alcoholic fatty liver disease; NASH, non-alcoholic steatohepatitis.

## Data Availability

The data are presented within the paper. Additional raw data or files are available on request from the corresponding author.

## References

[1] Bertuccio P., Turati F., Carioli G., Rodriguez T., La Vecchia C., Malvezzi M., Negri E. (2017). Global Trends and Predictions in Hepatocellular Carcinoma Mortality. J. Hepatol..

[2] Sung H., Ferlay J., Siegel R.L., Laversanne M., Soerjomataram I., Jemal A., Bray F. (2021). Global cancer statistics 2020: GLOBOCAN estimates of incidence and mortality worldwide for 36 cancers in 185 countries. CA Cancer J. Clin..

[3] Huang D.Q., El-Serag H.B., Loomba R. (2021). Global Epidemiology of NAFLD-Related HCC: Trends, Predictions, Risk Factors and Prevention. Nat. Rev. Gastroenterol. Hepatol..

[4] El-Serag H.B., Kanwal F. (2014). Epidemiology of Hepatocellular Carcinoma in the United States: Where Are We? Where Do We Go?. Hepatology.

[5] Nishikawa H., Kimura T., Kita R., Osaki Y. (2013). Treatment for Hepatocellular Carcinoma in Elderly Patients: A Literature Review. J. Cancer.

[6] El-Serag H.B., Rudolph K.L. (2007). Hepatocellular Carcinoma: Epidemiology and Molecular Carcinogenesis. Gastroenterology.

[7] Poon R.T., Fan S.T., Lo C.M., Liu C.L., Ngan H., Ng I.O., Wong J. (1999). Hepatocellular Carcinoma in the Elderly: Results of Surgical and Nonsurgical Management. Am. J. Gastroenterol..

[8] Reddy S.K., Barbas A.S., Turley R.S., Gamblin T.C., Geller D.A., Marsh J.W., Tsung A., Clary B.M., Lagoo-Deenadayalan S. (2011). Major Liver Resection in Elderly Patients: A Multi-Institutional Analysis. J. Am. Coll. Surg..

[9] Huang J., Li B.-K., Chen G.-H., Li J.-Q., Zhang Y.-Q., Li G.-H., Yuan Y.-F. (2009). Long-Term Outcomes and Prognostic Factors of Elderly Patients with Hepatocellular Carcinoma Undergoing Hepatectomy. J. Gastrointest. Surg..

[10] Takahashi H., Mizuta T., Kawazoe S., Eguchi Y., Kawaguchi Y., Otuka T., Oeda S., Ario K., Iwane S., Akiyama T. (2010). Efficacy and Safety of Radiofrequency Ablation for Elderly Hepatocellular Carcinoma Patients. Hepatol. Res..

[11] Song T., Lang M., Ren S., Gan L., Lu W. (2021). The Past, Present and Future of Conversion Therapy for Liver Cancer. Am. J. Cancer Res..

[12] Heimbach J.K., Kulik L.M., Finn R.S., Sirlin C.B., Abecassis M.M., Roberts L.R., Zhu A.X., Murad M.H., Marrero J.A. (2018). AASLD Guidelines for the Treatment of Hepatocellular Carcinoma. Hepatology.

[13] European Association for the Study of the Liver (2018). EASL Clinical Practice Guidelines: Management of Hepatocellular Carcinoma. J. Hepatol..

[14] Finn R.S., Qin S., Ikeda M., Galle P.R., Ducreux M., Kim T.-Y., Kudo M., Breder V., Merle P., Kaseb A.O. (2020). Atezolizumab plus Bevacizumab in Unresectable Hepatocellular Carcinoma. N. Engl. J. Med..

[15] Abou-Alfa G.K., Meyer T., Cheng A.-L., El-Khoueiry A.B., Rimassa L., Ryoo B.-Y., Cicin I., Merle P., Chen Y., Park J.-W. (2018). Cabozantinib in Patients with Advanced and Progressing Hepatocellular Carcinoma. N. Engl. J. Med..

[16] Kudo M., Finn R.S., Qin S., Han K.-H., Ikeda K., Piscaglia F., Baron A., Park J.-W., Han G., Jassem J. (2018). Lenvatinib versus Sorafenib in First-Line Treatment of Patients with Unresectable Hepatocellular Carcinoma: A Randomised Phase 3 Non-Inferiority Trial. Lancet.

[17] Zhu A.X., Park J.O., Ryoo B.-Y., Yen C.-J., Poon R., Pastorelli D., Blanc J.-F., Chung H.C., Baron A.D., Pfiffer T.E.F. (2015). Ramucirumab versus Placebo as Second-Line Treatment in Patients with Advanced Hepatocellular Carcinoma Following First-Line Therapy with Sorafenib (REACH): A Randomised, Double-Blind, Multicentre, Phase 3 Trial. Lancet Oncol..

[18] Bechis S.K., Carroll P.R., Cooperberg M.R. (2011). Impact of Age at Diagnosis on Prostate Cancer Treatment and Survival. J. Clin. Oncol..

[19] Adami H.O., Malker B., Holmberg L., Persson I., Stone B. (1986). The Relation between Survival and Age at Diagnosis in Breast Cancer. N. Engl. J. Med..

[20] Haymart M.R. (2009). Understanding the Relationship Between Age and Thyroid Cancer. Oncologist.

[21] Leff D.R., Chen A., Roberts D., Grant K., Western C., Windsor A.C.J., Cohen C.R.G. (2007). Colorectal Cancer in the Young Patient. Am. Surg..

[22] Guo H., Wu T., Lu Q., Dong J., Ren Y.-F., Nan K.-J., Lv Y., Zhang X.-F. (2017). Hepatocellular Carcinoma in Elderly: Clinical Characteristics, Treatments and Outcomes Compared with Younger Adults. PLoS ONE.

[23] Common Terminology Criteria for Adverse Events (CTCAE) v5.0. https://ctep.cancer.gov/protocoldevelopment/electronic_applications/ctc.htm#ctc_50.

[24] McGlynn K.A., Petrick J.L., London W.T. (2015). Global Epidemiology of Hepatocellular Carcinoma: An Emphasis on Demographic and Regional Variability. Clin. Liver Dis..

[25] Rich N.E., Yopp A.C., Singal A.G., Murphy C.C. (2020). Hepatocellular Carcinoma Incidence Is Decreasing Among Younger Adults in the United States. Clin. Gastroenterol. Hepatol..

[26] Oishi K., Itamoto T., Kobayashi T., Oshita A., Amano H., Ohdan H., Tashiro H., Asahara T. (2009). Hepatectomy for Hepatocellular Carcinoma in Elderly Patients Aged 75 Years or More. J. Gastrointest. Surg..

[27] Dohmen K., Shigematsu H., Irie K., Ishibashi H. (2003). Comparison of the Clinical Characteristics among Hepatocellular Carcinoma of Hepatitis B, Hepatitis C and Non-B Non-C Patients. Hepato-Gastroenterology.

[28] Mirici-Cappa F., Gramenzi A., Santi V., Zambruni A., Micoli A.D., Frigerio M., Maraldi F., Nolfo M.A.D., Poggio P.D., Benvegnù L. (2010). Treatments for Hepatocellular Carcinoma in Elderly Patients Are as Effective as in Younger Patients: A 20-Year Multicentre Experience. Gut.

[29] Liu P.-H., Hsu C.-Y., Lee Y.-H., Hsia C.-Y., Huang Y.-H., Su C.-W., Chiou Y.-Y., Lin H.-C., Huo T.-I. (2014). Uncompromised Treatment Efficacy in Elderly Patients With Hepatocellular Carcinoma: A Propensity Score Analysis. Medicine.

[30] Hepatozelluläres Karzinom. https://www.dgvs.de/wissen-kompakt/leitlinien/leitlinien-der-dgvs/hepatozellulaeres-karzinom/.

[31] Llovet J.M., Real M.I., Montaña X., Planas R., Coll S., Aponte J., Ayuso C., Sala M., Muchart J., Solà R. (2002). Arterial Embolisation or Chemoembolisation versus Symptomatic Treatment in Patients with Unresectable Hepatocellular Carcinoma: A Randomised Controlled Trial. Lancet.

[32] Biselli M., Forti P., Mucci F., Foschi F.G., Marsigli L., Caputo F., Ravaglia G., Bernardi M., Stefanini G.F. (1997). Chemoembolization versus Chemotherapy in Elderly Patients with Unresectable Hepatocellular Carcinoma and Contrast Uptake as Prognostic Factor. J. Gerontol. A Biol. Sci. Med. Sci..

[33] Mosconi C., Gramenzi A., Biselli M., Cappelli A., Bruno A., De Benedittis C., Cucchetti A., Modestino F., Peta G., Bianchi G. (2020). Survival and Tolerability of Transarterial Chemoembolization in Greater Versus Less than 70 Years of Age Patients with Unresectable Hepatocellular Carcinoma: A Propensity Score Analysis. Cardiovasc. Interv. Radiol..

[34] Yau T., Yao T.J., Chan P., Epstein R.J., Ng K.K., Chok S.H., Cheung T.T., Fan S.T., Poon R.T.P. (2009). The Outcomes of Elderly Patients with Hepatocellular Carcinoma Treated with Transarterial Chemoembolization. Cancer.

[35] Takayasu K., Arii S., Kudo M., Ichida T., Matsui O., Izumi N., Matsuyama Y., Sakamoto M., Nakashima O., Ku Y. (2012). Superselective Transarterial Chemoembolization for Hepatocellular Carcinoma. Validation of Treatment Algorithm Proposed by Japanese Guidelines. J. Hepatol..

[36] Llovet J.M., Ricci S., Mazzaferro V., Hilgard P., Gane E., Blanc J.-F., de Oliveira A.C., Santoro A., Raoul J.-L., Forner A. (2008). Sorafenib in Advanced Hepatocellular Carcinoma. N. Engl. J. Med..

[37] Cheng A.-L., Kang Y.-K., Chen Z., Tsao C.-J., Qin S., Kim J.S., Luo R., Feng J., Ye S., Yang T.-S. (2009). Efficacy and Safety of Sorafenib in Patients in the Asia-Pacific Region with Advanced Hepatocellular Carcinoma: A Phase III Randomised, Double-Blind, Placebo-Controlled Trial. Lancet Oncol..

[38] Raoul J.-L., Adhoute X., Penaranda G., Perrier H., Castellani P., Oules V., Bourlière M. (2019). Sorafenib: Experience and Better Manage ment of Side Effects Improve Overall Survival in Hepatocellular Carcinoma Patients: A Real-Life Retrospective Analysis. Liver Cancer.

[39] Marrero J.A., Kudo M., Venook A.P., Ye S.-L., Bronowicki J.-P., Chen X.-P., Dagher L., Furuse J., Geschwind J.-F.H., de Guevara L.L. (2016). Observational Registry of Sorafenib Use in Clinical Practice across Child-Pugh Subgroups: The GIDEON Study. J. Hepatol..

[40] Ogasawara S., Chiba T., Ooka Y., Kanogawa N., Motoyama T., Suzuki E., Tawada A., Kanai F., Yoshikawa M., Yokosuka O. (2014). Efficacy of Sorafenib in Intermediate-Stage Hepatocellular Carcinoma Patients Refractory to Transarterial Chemoembolization. Oncology.

[41] Arizumi T., Ueshima K., Minami T., Kono M., Chishina H., Takita M., Kitai S., Inoue T., Yada N., Hagiwara S. (2015). Effectiveness of Sorafenib in Patients with Transcatheter Arterial Chemoembolization (TACE) Refractory and Intermediate-Stage Hepatocellular Carcinoma. Liver Cancer.

[42] Park K.H., Kim J.H., Choe W.H., Kwon S.Y., Yoo B.C., Hwang J.H., Park S.W., Kim Y.J., Park H.S., Yu M.H. (2020). Risk Factors for Liver Function Deterioration after Transarterial Chemoembolization Refractoriness in Child-Pugh Class A Hepatocellular Carcinoma Patients. Korean J. Gastroenterol..

[43] Di Costanzo G.G., Tortora R., Iodice L., Lanza A.G., Lampasi F., Tartaglione M.T., Picciotto F.P., Mattera S., De Luca M. (2012). Safety and Effectiveness of Sorafenib in Patients with Hepatocellular Carcinoma in Clinical Practice. Dig. Liver Dis..

[44] Reig M., Torres F., Rodriguez-Lope C., Forner A., LLarch N., Rimola J., Darnell A., Ríos J., Ayuso C., Bruix J. (2014). Early Dermatologic Adverse Events Predict Better Outcome in HCC Patients Treated with Sorafenib. J. Hepatol..

[45] Tak K.Y., Nam H.C., Choi J.Y., Yoon S.K., Kim C.W., Kim H.Y., Lee S.W., Lee H.L., Chang U.I., Song D.S. (2020). Effectiveness of Sorafenib Dose Modifications on Treatment Outcome of Hepatocellular Carcinoma: Analysis in Real-Life Settings. Int. J. Cancer.

[46] Cochrane Library Geriatric Factors Predict Chemotherapy Feasibility: Ancillary Results of FFCD 2001-02 Phase III Study in First-Line Chemotherapy for Metastatic Colorectal Cancer in Elderly Patients. https://www.cochranelibrary.com/central/doi/10.1002/central/CN-00877144/full.

[47] Shibutani M., En W., Okazaki Y., Kashiwagi S., Fukuoka T., Iseki Y., Hirakawa K., Ohira M. (2021). The Efficacy and Safety of Trifluridine/Tipiracil Treatment for Elderly Patients With Metastatic Colorectal Cancer in a Real-World Setting. Anticancer Res..

[48] Kunzmann V., Siveke J.T., Algül H., Goekkurt E., Siegler G., Martens U., Waldschmidt D., Pelzer U., Fuchs M., Kullmann F. (2021). Nab-Paclitaxel plus Gemcitabine versus Nab-Paclitaxel plus Gemcitabine Followed by FOLFIRINOX Induction Chemotherapy in Locally Advanced Pancreatic Cancer (NEOLAP-AIO-PAK-0113): A Multicentre, Randomised, Phase 2 Trial. Lancet Gastroenterol. Hepatol..

[49] Nieß H., Kleespies A., Andrassy J., Pratschke P., Angele M.K., Guba M., Jauch K.-W., Bruns C.J. (2013). Pankreaskarzinom im hohen Alter. Chirurg.

[50] Feilhauer K., Hennig R., Lenz S., Köninger J. (2015). Pancreatic resection in the elderly: Is the risk justified?. Chir. Z. Alle Geb. Oper. Medizen.

